# Functioning and recovery during stroke rehabilitation: a comparison between pre-stroke frail and non-frail patients

**DOI:** 10.1007/s41999-023-00885-9

**Published:** 2023-11-07

**Authors:** Åsa Mennema, Thea P. M. Vliet Vlieland, Wilco P. Achterberg, Daniëlla M. Oosterveer, the SCORE-study group

**Affiliations:** 1 Basalt, Wassenaarseweg 501, 2333 AL Leiden, The Hague, The Netherlands; 2 Department of Orthopaedics, Rehabilitation and Physical Therapy, Leiden University Medical Center, Leiden, The Netherlands; 3 Department of Primary Care and Public Health, Leiden University Medical Center, Leiden, The Netherlands; 4 Department of Rehabilitation, Alrijne Hospital, Leiden, The Netherlands

**Keywords:** Stroke, Frailty, Rehabilitation, Outcomes

## Abstract

**Purpose:**

Pre-stroke frailty in older adults is associated with adverse outcomes after stroke in community-based and hospital-based populations. The aim of our study was to investigate the prevalence of pre-stroke frailty among older stroke survivors receiving medical specialistic rehabilitation and its association with outcomes and recovery.

**Methods:**

Pre-stroke frailty was measured by the Groningen Frailty Indicator (GFI, score ≥ 4 indicates frailty) in patients ≥ 65 years receiving stroke medical specialistic rehabilitation. Baseline, follow-up and change (i.e. recovery) scores of the Barthel index (BI), Stroke Impact Scale (SIS) ‘mobility’, ‘communication’, and ‘memory and thinking’, Hospital Anxiety and Depression Scale (HADS) and the EuroQoL-5 dimensions (EQ-5D) were compared between frail and non-frail patients with a multivariable regression model adjusting for confounders.

**Results:**

Of 322 included patients (34.2% females, median age 70 years), 43 (13.4%) patients reported pre-stroke frailty. There were no differences in BI or in destination of discharge between pre-stroke frail and non-frail stroke survivors receiving inpatient rehabilitation. However, pre-stroke frailty was associated with worse follow-up scores for all other measures. Recovery in pre-stroke frail patients was less favorable compared to non-frail patients for SIS mobility, HADS subscales and EQ-5D index and visual analogue scale.

**Conclusion:**

Pre-stroke frailty was present in a minority of older stroke survivors receiving medical specialistic rehabilitation. BI and destination of discharge did not differ. Nevertheless, pre-stroke frailty was associated with worse functioning at follow-up for most measures of health status and with smaller improvements in mobility, mood and quality of life.

## Key summary points

**Aim:**

Investigate the prevalence of pre-stroke frailty among older stroke survivors receiving medical specialistic rehabilitation and its association with outcomes and recovery.

**Findings:**

Pre-stroke frailty was associated with worse functioning at follow-up for most measures of health status and with smaller improvements in mobility, mood and quality of life.

**Message:**

More research is of value to assess the role of pre-stroke frailty as an instrument to help allocating stroke patient to the most suitable rehabilitation.

## Introduction

Stroke is worldwide a leading cause of disabilities acquired as an adult [1]. Its incidence is increasing because of the aging population [2]. In addition, the prevalence is increasing because of improved survival rates due to better acute stroke treatment [3]. As a consequence, more stroke-surviving patients are older adults, including frail older adults.

Frailty is defined by The World Health Organization and ADVANTAGE Joint Action of the European Union as ‘a progressive age-related decline in physiological systems that results in decreased reserves of intrinsic capacity, which confers extreme vulnerability to stressors and increases the risk of a range of adverse health outcomes’ [4]. Frailty is a multidimensional concept that takes biological, physiological, social, cognitive and emotional aspects into account [5, 6].

Frailty has a bidirectional link with a stroke: frail older adults have a higher risk of having a stroke, and stroke patients have a higher risk on becoming frail [7]. In addition, previous studies demonstrated that pre-stroke frailty was associated with worse outcomes of stroke: lower survival, lower National Institutes of Health Stroke Scale (NIHSS) score, more complications, more cognitive decline and more limitations of daily activities [8–14].

These associations were found in community-based and hospital-based stroke populations. Research in rehabilitation-based populations is scarce. In The Netherlands, after acute inhospital treatment, stroke survivors can be referred to their home, a nursing home or medical specialistic rehabilitation or geriatric rehabilitation. Regarding the latter two, compared with geriatric rehabilitation, stroke patients referred to medical specialistic rehabilitation are usually younger and were more active before their stroke [15]. However, it is unclear whether and to what extent patients referred to medical specialist rehabilitation are frail. In addition, it is unknown if pre-stroke frailty is also associated with worse outcomes in patients receiving this stroke rehabilitation. More knowledge on the presence and role of frailty might help in allocating patients to the optimal rehabilitation setting and in predicting rehabilitation outcomes. Based on previous research, we hypothesized that pre-stroke frailty in older stroke survivors receiving medical specialistic rehabilitation is associated with more disabilities at baseline and during follow-up, and with less recovery (defined as the change score between baseline and follow-up). The aim of this study was to investigate whether pre-stroke frail patients were present in a medical specialistic rehabilitation setting and whether or not our hypothesis is true: is pre-stroke frailty related to functioning at start of rehabilitation and during follow-up and to recovery in patients of 65 years or older receiving stroke rehabilitation?

## Methods

### Setting

In the Netherlands, there were more than 48.500 admissions to the hospital because of stroke in 2020 [16]. After hospital discharge 71% of patients were discharged to home and 29% were referred to an inpatient rehabilitation setting [17]. Of these inpatients, 48% receive inpatient medical specialistic rehabilitation and 52% receive geriatric rehabilitation [17]. The indication for medical specialistic rehabilitation is set by a rehabilitation physician, in general, for younger and pre-stroke active patients [15]. In addition, part of the patients that go home receive outpatient medical specialistic rehabilitation.

### Design

The Stroke Cohort Outcomes of REhabilitation (SCORE) study is an observational, prospective cohort study, which started on March 10, 2014 (Netherlands Trial Registry no. 4292) [18]. In this study, data were collected from stroke patients who received inpatient and/or outpatient multidisciplinary rehabilitation at Basalt, a facility that delivers medical specialistic rehabilitation. The study protocol was approved by the Medical Ethics Committee of the Leiden University Medical Center (P13.249) [18]. All patients signed informed consent before participation in this study. Study results are reported in accordance with the Strengthening the Reporting of Observational studies in Epidemiology (STROBE) guidelines [19].

### Patients

Consecutive stroke patients were included in the SCORE study when they were diagnosed with stroke in the previous six months or less, and aged ≥ 18 years. Patients were excluded when they were diagnosed with dementia or a psychiatric disorder or when they were unable to complete questionnaires in Dutch.

For this research question, we selected patients who were 65 year or older at the time of stroke, and who completed the GFI.

### Sociodemographic and clinical characteristics

Age, sex, type of stroke, the number of days between stroke and the start of rehabilitation, the duration of inpatient rehabilitation, outpatient rehabilitation after discharge (yes/no) and destination of discharge were extracted from the medical files. Level of education (low, middle or high level) and living situation (living alone or with others) were collected through a standardized questionnaire at the start of the rehabilitation, i.e. baseline. Comorbidities were assessed at baseline by the Dutch Life Situation Cohort Questionnaire comprising 16 chronic diseases, including diabetes mellitus and hypertension [20].

### Pre-stroke frailty

Pre-stroke frailty was measured at baseline with the GFI, a patient-reported outcome measure (PROM) [21]. The GFI was completed by patients themselves with the instruction to report the situation before the stroke; a caregiver was allowed to help the patient. The GFI comprises 15 items. Each answer is dichotomized into 0 or 1, where 1 indicates a problem or dependency, leading to a sum score ranging from 0 to 15 points [21]. At least 75% of the GFI should be completed to calculate a sum score [22]. A higher score indicates more frailty and a sum score of four points or higher indicates a frail patient. GFI was shown to have three subscales, i.e. Daily activities (items 1–4), Health problems (items 5–10) and Psychosocial functioning (items 11–15) [23]. Previously, the GFI was shown to have adequate feasibility, internal consistency and construct validity in older adults [22, 24].

### Measurements of functioning and recovery

Functioning was measured with Dutch versions of the Barthel Index (BI), the domains ‘Mobility’, ‘Communication’ and ‘Memory and thinking’ of the Stroke Impact Scale (SIS), the Hospital Anxiety and Depression Scale (HADS) and the EuroQoL 5 Dimensions (EQ5D). Recovery was defined as a change of score between baseline and discharge for the Barthel Index. For all other measures, recovery was defined as a change of score between baseline and six months thereafter (i.e. follow-up).

The BI measures functional dependence for basic activities of daily life, with a score ranging from 0 (i.e. totally dependent) to 20 (i.e. totally independent) [25]. The BI was completed only for inpatients by a nurse at baseline and at discharge from the rehabilitation center.

The SIS is a stroke-specific PROM, that assesses several domains [26, 27]. Scores for each SIS domain range from 0 to 100, with higher scores indicating better functioning on that specific domain. The SIS domain ‘Communication’ and ‘Memory and thinking’ were administered to all patients. In April 2015, the SIS domain ‘Mobility’ was added to the SCORE study. The SIS domain ‘Mobility’, ‘Communication’ and ‘Memory and thinking’ was completed by patients at baseline, and six months thereafter.

The HADS was used to measure anxiety and depression symptoms and consists of 14 items [28]. This PROM leads to a score for anxiety symptoms (HADS-A) and a score for depression symptoms (HADS-D). Each sub score can range from 0 to 21, where higher scores denote more symptoms and a score of 8 or higher indicates a possible anxiety disorder or depression, respectively [29, 30]. The HADS was completed by patients at baseline, and six months thereafter.

The EQ5D, version EQ5D 3 Levels, was used to measure health-related quality of life [31]. In this standardized instrument, patients rate their health on five dimensions (mobility, self-care, usual activities, pain/discomfort and anxiety/depression). Each dimension had three levels of severity. This leads to a EQ5D index, with a score ranging from -0.33 (serious problems on all five dimensions) to 1 (healthy). Next to the index, the EQ5D comprises a vertical visual analogue scale (VAS), that is used as a quantitative measure of overall health status [31]. The EQ5D was completed by patients at baseline, and six months thereafter.

### Statistical analyses

Data were anonymized when entered into a database and were analyzed with IBM SPSS 24.0 for Windows. A two-sided *p* value of 0.05 was considered statistically significant.

Data are presented as numbers (*N*) with percentages (%), medians with interquartile ranges (IQR) or means with standard deviations (SD), depending on the nature of the variables and their distribution. The Kolmogorov–Smirnov test was performed to assess whether or not continuous variables were normally distributed. No imputation was used for missing data.

Age and sex of included patients were compared with patients who were excluded because they did not complete the GFI using the Fisher exact test or the Mann–Whitney *U* test.

For included patients, GFI answers and subscales, sociodemographic and clinical characteristics were compared between frail patients (GFI ≥ 4) and non-frail patients (GFI < 4) using the Fisher’s exact test or the Mann–Whitney *U* test.

Recovery for the Barthel Index was calculated by subtracting the baseline score from the score at discharge. Recovery for the SIS domains ‘communication’ and ‘mobility’, HADS-A, HADS-D, EQ5D index and VAS was calculated by subtracting baseline scores from follow-up scores six months after baseline. Scores at baseline and follow-up and recovery score were compared between frail patients and non-frail patients with the Mann–Whitney *U* test. For the HADS-A and the HADS-D, we also analysed differences in the dichotomized scores (< 8 and ≥ 8) between frail and non-frail patients with the Fisher’s Exact test.

In addition, recovery scores were analysed with multivariable linear regression analyses adjusted for age, sex, the number of days between stroke and the start of the rehabilitation and baseline scores (dependent: recovery score; independent: pre-stroke frailty (yes/no).

## Results

Between March 2014 and December 2019, 836 stroke patients were included in the SCORE study. Of these patients, 462 (55.3%) patients were excluded because they were younger than 65 years old; an additional 52 (6.2%) patients were excluded because they did not complete the GFI. The remaining 322 (38.5%) patients were included in the current analyses. The age and sex of these included patients did not significantly differ from those of the patients that did not complete the GFI (median age of 70 years (IQR 68–74) versus 70 (IQR 66–73), *p* = 0.275; 34.2% females versus 34.6%, *p* = 1.000).

The median GFI of the included patients was 1 with a range of 0 to 10. There were 113 (35.1%) patients with a GFI of 0. There were 43 (13.4%) patients with a sum score of four points or higher and were therefore annotated as pre-stroke frail patients.

### Characteristics of pre-stroke frail and non-frail patients

The characteristics of pre-stroke frail and non-frail patients are shown in Table 1. The diagnosis of recurrent stroke was more frequent in frail than in non-frail patients (33.3% versus 8.0%, *p* = 0.001). Frail patients were more likely to live alone than non-frail patients (45.2% versus 27.5%, *p* = 0.029) and had a higher number of comorbidities (median number of comorbidities of 2 (IQR 2–4, mean 2.6) versus 2 (IQR 1–2, mean 1.9), *p* = 0.006). There were no other significant differences in characteristics between both groups. More specifically, there were no differences in the rehabilitation starting site nor in the destination of discharge.

**Table 1:** Characteristics of pre-stroke frail and non-frail patients receiving stroke rehabilitation

	Frail patients		Non-frail patients		*p* value
	*N*	*N* total = 43	*N*	*N* total = 279	
Age (years)	42	70 (67–74)	279	70 (68–74)	0.852
Female sex	43	14 (32.6%)	279	96 (34.4%)	0.864
Ischemic stroke	42	36 (85.7%)	277	231 (83.4%)	0.825
Recurrent stroke	27	9 (33.3%)	162	13 (8.0%)	**0.001**
Education	42		275		0.926
Low		23 (54.8%)		140 (50.9%)	
Middle		7 (16.7%)		52 (18.9%)	
High		12 (28.6%)		83 (30.2%)	
Living alone pre-stroke	42	19 (45.2%)	276	76 (27.5%)	**0.029**
Comorbidity^a^	31	2 (2–4)	215	2 (1–2)	**0.006**
Diabetes mellitus	42	9 (21.4%)	276	58 (21.0%)	1.000
Hypertension	42	28 (66.7%)	271	144 (53.1%)	0.133
Number of days between stroke and start of rehabilitation	39	29 (19–39)	275	27 (19–40)	0.758
Start with inpatient rehabilitation	43	37 (86.0%)	279	244 (87.5%)	0.806
Duration of inpatient rehabilitation in days^b^	35	43 (30–68)	236	42 (27- 65)	0.466
Continuation with outpatient rehabilitation^b^	37	16 (43.2%)	244	92 (37.7%)	0.587
Destination of discharge^b^	39		237		
Home		36 (92.3%)		229 (96.6%)	0.129
Nursing home		2 (5.1%)		3 (1.3%)	
Hospital		1 (2.6%)		5 (2.1%)	

Frail is defined as a score ≥ 4 on Groningen Frailty Indicator. Data are presented as numbers (*N*) with percentages (%) or medians with interquartile ranges (IQR). The *p* values are the result of the Fisher exact test or the Mann–Whitney *U* test, *p* < 0.05 are annotated in bold

^a^Measured with the Dutch Life Situation Cohort Questionnaire

^b^For inpatients only

### The GFI

For all items, except for memory, the proportions of patients with a score of 1 were statistically significantly higher in frail than in non-frail patients.

### Baseline and follow-up scores

In Table 2, baseline and follow-up scores of the Barthel Index, SIS Mobility, SIS Communication, SIS Memory and thinking, HADS-A, HADS-D, EQ5D index and VAS are shown for both pre-stroke frail and non-frail patients. Regarding the baseline scores, pre-stroke frail patients had worse scores on the SIS Communication and Memory and thinking subscales, on the HADS-A and HADS-D, and on the EQ-5D index and VAS (all* p* < 0.01), whereas there were no differences in Barthel Index and SIS Mobility. Similar results were seen at follow-up, where the lower SIS Mobility scores seen in pre-stroke frail patients compared to non-frail also reached statistical significance.

**Table 2:** Measurements of functioning at baseline and during follow-up and of recovery in pre-stroke frail and non-frail patients receiving stroke rehabilitation

	Frail patients		Non-frail patients		*p* value^a^	*p* value^b^
	*N*	*N* total = 43	*N*	*N* total = 279		
**Barthel Index**^**c**^						
Baseline	27	17 (11–18)	186	16 (11–18)	0.769	
At discharge	24	20 (19–20)	151	20 (19–20)	0.890	
Recovery	23	3 (2–8)	143	3 (1–7)	0.610	0.862
**SIS mobility**						
Baseline	32	65 (44–96)	169	81 (54–94)	0.254	
At 6 months	23	75 (47–94)	141	92 (79–100)	**0.005**	
Recovery	23	6 (− 3 to 33)	134	6 (0–25)	0.696	**0.024**
**SIS communication**						
Baseline	41	85 (73–93)	263	93 (80–100)	**0.003**	
At 6 months	32	86 (76–93)	221	93 (86–100)	**0.001**	
Recovery	31	0 (− 6 to 7)	210	0 (− 4 to 7)	0.580	0.076
**SIS memory and thinking**						
Baseline	42	79 (61–94)	264	89 (75–99)	**0.002**	
At 6 months	32	80 (71–93)	223	92 (79–100)	**0.002**	
Recovery	32	4 (− 4 to 11)	212	0 (− 4 to 7)	0.339	0.886
**HADS-A**						
Baseline	31	7 (5–10)	165	3 (2–6)	**< 0.001**	
At 6 months	29	9 (6–12)	220	3 (1–5)	**< 0.001**	
Recovery	21	0 (− 3 to 3)	132	− 1 (− 2 to 1)	0.242	**< 0.001**
**HADS-D**						
Baseline	31	7 (4–10)	164	3 (1–6)	**< 0.001**	
At 6 months	29	8 (5–12)	221	3 (1–6)	**< 0.001**	
Recovery	21	1 (− 1 to 4)	132	0 (− 2 to 1)	**0.012**	**< 0.001**
**EQ-5D index**						
Baseline	43	0.68 (0.39–0.81)	250	0.81 (0.64–0.89)	**< 0.001**	
At 6 months	30	0.66 (0.29–0.81)	218	0.85 (0.78–1.00)	**< 0.001**	
Recovery	30	0.00 (− 0.10 to 0.26)	197	0.03 (− 0.00 to 0.19)	0.333	**< 0.001**
**EQ-5D VAS**						
Baseline	43	59 (40–71)	261	70 (55–80)	**0.006**	
At 6 months	31	61 (40–71)	219	75 (65–85)	**< 0.001**	
Recovery	31	0 (− 10 to 15)	208	5 (− 3 to 19)	0.317	**< 0.001**

Frail is defined as a score ≥ 4 on Groningen Frailty Indicator. Data are presented as medians with interquartile ranges (IQR). *p*< 0.05 are annotated in bold﻿

*HADS-A* Hospital Anxiety and Depression Scale anxiety subscale; *HADS-D* Hospital Anxiety and Depression Scale depression subscale; *SIS* stroke impact scale

^a^The *p* values are the result of the Mann–Whitney *U* test

^b^The p values are the result of multivariable regression analyses adjusted for age, sex, the number of days between stroke and start of rehabilitation and baseline scores﻿

^c^For inpatients only

In line with the ordinal scale analyses of the HADS, the dichotomic analyses of the HADS demonstrated that the proportion of patients with a possible anxiety disorder or depression was higher in pre-frail patients than in non-frail. There were 13/31 (41.9%) pre-stroke frail patients with a possible anxiety disorder versus 20/164 (12.1%) non-frail patients at baseline (*p* < 0.001), and 18/29 (62.1%) versus 23/220 (10.5%) at follow-up, respectively (*p* < 0.001). As for possible depression, there were 14/31 (45,2%) pre-stroke frail patients versus 26/164 (15.9%) non-frail patients at baseline (*p* = 0.001), and 17/29 (58.6%) versus 30/221 (13.6%) at follow-up, respectively (*p* < 0.001).

### Recovery

The crude comparison of the recovery scores with the Mann Whitney-*U* (Table 3) showed that the changes for all measures were not different between the two groups, with the exception of recovery for depressive symptoms (HADS-D). Depressive symptoms as measured with the HADS-D resolved less in pre-stroke frail patients than in non-frail patients (recovery 1 (IQR − 1 to 4) versus 0 (IQR − 2 to 1), *p* = 0.012).

**Table 3:** Results of multivariable regression analyses adjusted for age, sex, the number of days between stroke and start of rehabilitation and baseline scores

Recovery score of (dependent)	*β* for pre-stroke frailty (independent)	95% CI	*p* value
Barthel index^a^	− 0.08	− 1.04 to 0.88	0.862
SIS mobility	− 8.09	− 15.08 to − 1.10	**0.024**
SIS communication	− 4.04	− 8.50 to 0.42	0.076
SIS memory and thinking	− 0.35	− 4.49 to 5.19	0.8804
HADS-A	4.10	2.47–5.73	**< 0.001**
HADS-D	4.07	2.38–5.77	**< 0.001**
EQ-5D index	− 0.15	− 0.22 to − 0.08	**< 0.001**
EQ-5D VAS	− 12.21	− 18.20 to -6.21	**< 0.001**

For the HADS (higher score, more anxiety or depressive symptoms), a *positive* *β* annotates less recovery for pre-stroke frail patients. For all other measures (higher score, better functioning), a *negative* *β* annotates less recovery for pre-stroke frail patients. No imputation of missing data was performed. *p* < 0.05 are annotated in bold﻿

*HADS-A* Hospital Anxiety and Depression Scale anxiety subscale, *HADS-D* Hospital Anxiety and Depression Scale depression subscale, *SIS* stroke impact scale

^a^For inpatients only

Adjusted for age, sex, the number of days between stroke and start of rehabilitation and baseline scores, similar results were found for the Barthel Index, the SIS communication, and for the SIS memory and thinking: there was no statistically significant difference in recovery (Table 3). However, recovery in pre-stroke frail patients was significantly worse for SIS mobility, HADS-A, HADS-D, EQ5D index and EQ5D VAS.

## Discussion

Our results demonstrate that patients with pre-stroke frailty are present within a stroke population receiving medical specialistic rehabilitation, but in a minority of patients, namely 13.4%. Frail patients had more comorbidities, lived more alone and rehabilitated more for a recurrent stroke, but the destination of discharge did not differ between pre-stroke frail and non-frail inpatients, Pre-stroke frail patients and non-frail patients reported no difference in baseline mobility. Nor were differences found in baseline and follow-up activities of daily life as measured with the Barthel Index Nevertheless, pre-stroke patients reported worse functioning in mobility during follow-up, in communication, memory and thinking, and quality of life. In addition, they reported more anxiety and depressive symptoms. When looking at recovery, pre-stroke frail patients showed worse recovery for mobility and quality of life compared to non-frail patients. Moreover, anxiety and depressive symptoms in pre-frail patients diminished less than in non-frail patients.

The prevalence of pre-stroke frailty of 13.4% is rather low compared to most previous literature: in recent literature a study of Yang et al. [12] found a higher prevalence of 21.4% of pre-stroke frailty among older persons with an acute stroke using the FRAIL scale [32] and a review of Burton et al. [33] found a pooled prevalence of pre-stroke frailty of 24.6%. Only one study, of Kanai et al. [10], found a similar prevalence of 12.4% pre-stroke frailty among older persons with an acute stroke using the Frailty Screening Index [34]. The comparison with other studies is, however, difficult because apart from various settings, different measures of frailty can lead to very different results in prevalence: Drubbel et al. [11] found in the patients aged ≥ 60 years in a Dutch primary care center a prevalence of frailty of 39.1% using the GFI and a prevalence of 60.0% using the Frailty Index. Hanlon et al. [14] found a prevalence of 23.8% with the frailty phenotype, 30.1% with the clinical frailty scale and even higher prevalence using the Frailty Index. These differences in the prevalence of frailty in the same population using different measures implicates that our findings might have been different when a different measure was used. A different measure could lead to an even better or worse selection of those patients that recover less. To assess which measure of frailty is best in this selection, more research is necessary.

Pre-stroke frailty measured with the GFI was previously found more often in patients who were older, who lived alone and were less often highly educated in a primary care stroke population [11] Our results confirmed the association between pre-stroke frailty and living alone. The other associations were not found; this could be due to the difference in population or the small number of pre-stroke frail patients in our study that did not allow us to detect these associations.

Frailty in patients of 65 years or older receiving stroke rehabilitation was not related to worse recovery on all outcomes in contrast to our hypothesis. Although frailty was associated with worse recovery for most outcome measures, this was not seen for the Barthel Index. Here it must be noted that the Barthel Index was only assessed in inpatient patients. In addition, a previously reported ceiling effect of the Barthel Index might explain the absence of differences at discharge and recovery [35]. Indeed, in our population: 75.0% of pre-frail patients and 74.2% of non-frail patients had the maximum score of 20.

The patients included in this study had an indication for medical specialistic rehabilitation by a rehabilitation physician were included in our population. This indication is based on an estimation that patients will benefit from this stroke rehabilitation. This might have led to a selection of the ‘relatively good’ frail patients. The presence of a possible selection bias is indeed suggested by the relatively low prevalence of pre-stroke frailty in our study as described above [12, 33].

In line with our results, a meta-analysis showed a high prevalence of depression (i.e. 38.60%) among frail persons [36]. This meta-analysis also demonstrated that depression increases the risk of frailty, and the authors suggest that these reciprocal associations might be explained by shared risk factors and pathophysiological pathways [37]. As for treatment, evidence for psychological treatment for depression in frail patients is scarce; there is only low quality of evidence that this treatment is effective in older adults with no studies examining specifically frail patients [37]. Other studies found an attenuated response to antidepressant medication in frail patients [38, 39]. Our results showed that depressive symptoms in frail patients remain high, even though different therapists including psychologists treat the patient. These results and the previous literature suggest there is a need for optimizing treatment for depression in this specific subgroup.

In addition, the association of frailty with anxiety symptoms was found in community-dwelling older adults and in patients with atrial fibrillation, in line with our results [40, 41]. Similar to depressive symptoms, our results demonstrate that anxiety symptoms remained high during rehabilitation, and treatment optimization seems necessary.

The strength of our study is the prospective design of the study and that this study is the first to examine pre-stroke frailty in a rehabilitation population and its influence on several different outcomes. Both clinical measures and patient-reported measures are used, with a follow-up up to six months after the start of rehabilitation. During this follow-up duration most recovery will have taken place [42].

As for study limitations: we had no data about stroke severity measured with the mRS or NIHSS, while stroke severity was found a strong predictor of recovery [30]. In our analyses, we have tried to correct for this by adding baseline scores of the measurements to the multivariable regression analyses. Next to this, patients were excluded from inclusion, when they were severely affected (severe aphasia, severe cognitive problems, severe motor problems) and therefore could not complete questionnaires. We have no information on whether pre-stroke frailty was more common among these patients, but this is a real possibility since the NIHSS score was previously found to be associated with pre-stroke frailty [10].

In addition, there were missing data that were not due to measuring the outcome in a subset of patients (i.e. the Barthel Index only in inpatients) or due to adding a PROM after the first period of inclusion was done (i.e. SIS Mobility). These missing data could potentially affect our results. On the other hand, our population included a relatively small number of frail patients. Therefore, not all differences in functioning or recovery might have been found by our study.

Another limitation was that only pre-stroke frailty was measured: a follow-up six months after baseline would have allowed us to investigate how many stroke patients in a rehabilitation-based population had become frail.

In conclusion, in general pre-stroke frailty was although relatively uncommon, associated with worse outcomes in stroke patients receiving medical specialistic rehabilitation. Recovery in pre-stroke frail patients was less favorable compared to non-frail patients for mobility, mood and quality of life. However, this was not true for activities of daily life, nor for total days of admission and destination at discharge of inpatients, indicating that pre-stroke frailty does not have to be an absolute exclusion criterium for medical specialistic rehabilitation.

These results demonstrate that more research is of value to assess the role of pre-stroke frailty as an instrument to help allocating stroke patient to the most suitable rehabilitation. In addition, there is a need for effective interventions for both depressive and anxiety symptoms in pre-frail stroke patients so that stroke rehabilitation can be optimized for this specific group of patients.
